# Assessment of acetabular chondral damage and labral pathologies via direct MR arthrography: specialization matters

**DOI:** 10.1007/s00402-021-04071-z

**Published:** 2021-07-19

**Authors:** A. Zimmerer, MM. Schneider, K. Tramountanis, V. Janz, W. Miehlke, GI. Wassilew, C. Sobau

**Affiliations:** 1 ARCUS Sportklinik, Rastatter str, 17-19, 75179 Pforzheim, Germany; 2 Department of Orthopedics and Orthopedic Surgery, University Medicine Greifswald, Ferdinand-Sauerbruch-Straße, 17475 Greifswald, Germany; 3 University of Witten/Herdecke, Alfred-Herrhausen-Straße 50, 58455 Witten, Germany

**Keywords:** FAI syndrome, Hip joint, Acetabular labrum, Chondral damage, Magnet resonance imaging

## Abstract

**Aims:**

To compare the diagnostic accuracy of investigators from different specialities (radiologists and orthopaedic surgeons) with varying levels of experience of 1.5 T direct magnetic resonance arthrography (dMRA) against intraoperative findings in patients with femoroacetabular impingement syndrome (FAIS).

**Methods:**

A total of 272 patients were evaluated with dMRA and subsequent hip arthroscopy. The dMRA images were evaluated independently by two non-hip-arthroscopy-trained orthopaedic surgeons, two fellowship-trained musculoskeletal radiologists, and two hip-arthroscopy-trained orthopaedic surgeons. The radiological diagnoses were compared with the intraoperative findings.

**Results:**

Hip arthroscopy revealed labral pathologies in 218 (79%) and acetabular chondral lesions in 190 (69%) hips. The sensitivity, specificity, positive predictive value (PPV), negative predictive value (NPV) and accuracy for evaluating the acetabular labral pathologies were 79%, 18%, 79%, 18%, and 66% (non-hip-arthroscopy trained orthopaedic surgeons), 83%, 36%, 83%, 36%, and 74% (fellowship-trained musculoskeletal radiologists), and 88%, 53%, 88%, 54% and 81% (hip-arthroscopy trained orthopaedic surgeons). The sensitivity, specificity, PPV, NPV and accuracy of dMRA for assessing the acetabular chondral damage were 81%, 36%, 71%, 50%, and 66% (non-hip-arthroscopy trained orthopaedic surgeons), 84%, 38%, 75%, 52%, and 70% (fellowship-trained musculoskeletal radiologists), and 91%, 51%, 81%, 73%, and 79% (hip-arthroscopy trained orthopaedic surgeons). The hip-arthroscopy trained orthopaedic surgeons displayed the highest percentage of correctly diagnosed labral pathologies and acetabular chondral lesions, which is significantly higher than the other two investigator groups (*p* < 0.05).

**Conclusion:**

The accuracy of dMRA on detecting labral pathologies or acetabular chondral lesions depends on the examiner and its level of experience in hip arthroscopy. The highest values are found for the hip-arthroscopy-trained orthopaedic surgeons.

**Level of evidence:**

Retrospective cohort study; III.

## Introduction

Femoroacetabular impingement syndrome (FAIS) is one of the main causes of groin pain in younger patients and leads to symptomatic osteoarthritis over time [1]. Hip arthroscopy (HA) has established itself as the gold standard in the treatment of FAIS [2–6]. Currently, direct magnetic resonance arthrography (dMRA) is the more commonly used, and more sensitive, imaging modality for detecting intraarticular pathologies compared to conventional magnetic resonance imaging (cMRI) [7–10]. However, false-positive evaluation can occur in up to 20% of cases with labral pathologies being the main reason for misinterpretation [11]. Additionally, HA often reveals additional, previously undetected pathologies in the dMRA, intraoperatively [6, 11–13].

The interobserver reliability for cMRI and dMRA has been previously investigated for either radiologists or orthopaedic surgeons, but never both [10, 14–17]. Usually, the studies and examinations were carried out by radiologists or orthopaedic surgeons. It has been shown for example that musculoskeletal radiologists perform better than community radiologists in terms of overall accuracy in radiological reporting of hip pathology [18]. So far, no distinction was made in any study between radiologists and orthopaedic surgeons regarding the hip joint. However, the experience of a specialized orthopedic surgeon may have a significant impact on the evaluation of pathologic MRIs [19]. Therefore, it was the aim of this study to compare the diagnostic accuracy of reading the dMRA between a non-HA trained surgeon, a radiologist and a fellowship-trained HA surgeon against to the gold standard of hip arthroscopy. We hypothesize, that a fellowship-trained HA surgeon would be able to detect acetabular chondral damage and labral pathologies with a higher accuracy in dMRA than a non-HA-trained orthopaedic surgeon and a fellowship-trained musculoskeletal radiologist.

## Methods

This was a single-center retrospective cohort study. After gaining approval from the local ethics committee, the hospital registry was reviewed to identify all patients who received a hip dMRA and subsequent arthroscopic therapy for FAIS at our institution between January 2014 and December 2015. The inclusion criteria were primary HA for the treatment of FAIS, patient age > 18 years, and dMRA using a standardized protocol as defined below. The exclusion criteria were HA for a pathology other than FAIS, revision-surgery, patient age < 18 years or magnetic resonance (MR) examination contrary to the following standard MR protocol.

### Direct MR arthrography protocol

The hip dMRAs were performed with a 1.5 T MR Scanner (either Avanto or Symphony, Siemens Healthcare, Erlangen, Germany) using an 18-channel flex torso array coil (Siemens Healthcare, Erlangen, Germany) around the patient, centered over the affected hip. The hip was punctured under the guidance of ultrasound, and then 15–20 ml of Gadopentetate–Dimeglumine 2 mmol/l (Magnevist, Bayer Vital GmbH, Leverkusen, Germany) were injected. After injection MR imaging was obtained. The total examination time was 33 ± 3 (29–237) minutes on average.

A summary of the dMRA hip protocol and parameters used at our institution can be found in Table 1. Radial slices rotating around the femoral head–neck axis were performed according to the 3D reconstructions, as radial planes are extremely helpful in detecting labral pathologies.

**Table 1:** Routine dMRA hip sequences and parameters for 1.5 T MR scanners

Sequence	Plane	Slice thickness (mm)	TR (ms)	TE (ms)	Field of view (mm^2^)	Acquisition matrix
T1 SE	Coronal	3	480	15	200	512 × 512
T1 TSE	Sagittal	4	786	12	200	512 × 512
T2 TIRM	Coronal	4	4640	59	200	256 × 256
T2 DE3D	Coronal	1.46	42	43	190	256 × 256
T2 DE3D	Sagittal	1.7	23.56	6.533	180	320 × 320
PD TSE FS	Coronal	3	2210	36	200	256 × 256
PD TSE FS	Transversal	4	2470	36	200	256 × 256

*DE3D* dual echo three-dimensional, *FS* fat suppressed, *dMRA* direct magnetic resonance arthrography, *PD* proton density, *TE* echo time, *TIRM* Turbo inversion recovery magnitude, *TR* repetition time, *TS* spin echo, *TSE* turbo spin echo

### Surgical technique

All procedures were performed by a senior HA-trained orthopaedic specialist, who performs more than 200 hip arthroscopies per year. HA was performed in a supine position under general anesthesia. The patients were positioned on a traction table with a well-padded perineal post and subjected to traction to distract the hip. In total, 2–3 standard arthroscopy portals were used, depending upon if labral repair was performed during the surgery: the anterolateral and midanterior portal, were always used, and the distal anterolateral accessory portal was utilized if labral repair was necessary [20]. A routine evaluation of the joint was then undertaken to evaluate the labrum and articular chondral status. The acetabular cartilage was graded using the acetabular labrum articular disruption (ALAD) classification [21]. Labral pathologies were classified according to Beck’s classification [22]. A capsulotomy was performed between the anterolateral and midanterior regions, if necessary. Loop- or base-repair techniques were used to repair labral tears in case the labrum showed acceptable consistency and quality [23]. If the labrum was no longer repairable, selective labral debridement was performed while preserving as much of the stable labrum as possible to retain a functional seal between the labrum and the femoral head. Chondral lesions were treated by chondroplasty or abrasion according to the different damage stages. In the case of focal anterior pincer morphology, the anterior rim was carefully trimmed using a round 4 mm burr. Femoroplasty was performed if cam morphology was present. The capsule was not routinely closed, as there was not much evidence for capsular closure available during the eligibility period.

### Direct MR arthrography evaluation

All dMRA studies were retrospectively reviewed by two non-HA-trained orthopaedic surgeons (investigator group 1), two fellowship-trained musculoskeletal radiologists (investigator group 2), and two HA-trained orthopaedic surgeons (investigator group 3). Each of the HA-trained orthopaedic surgeons performs more than 250 hip arthroscopies per year and each of the fellowship-trained musculoskeletal radiologists diagnoses more than 200 dMRAs of the hip per year. All investigators were blinded to the arthroscopic findings and patients’ clinical information. All investigators were not involved in the HA. Each of the investigators analyzed all dMRA images and conducted each assessment twice. Labral pathologies were assessed using the Czerny classification [8]. Chondral lesions were assessed using Outerbridge classification [24].

### Statistical analysis

Data were analyzed using SPSS version 22 (IBM, Armonk, NY). Analysis of the dMR arthrography assessment of labral pathologies and acetabular chondral lesions were referenced against the gold-standard HA. Intraobserver reliability for each investigator group was evaluated using the intraclass correlation coefficient (ICC). Reliability was scored: very good (0.81–1), good (0.61–0.8), moderate (0.41–0.6), fair (0.21–0.4) or poor (< 0.2) [25]. Sensitivity, specificity, positive and negative predictive values (PPV, NPV), and accuracy were calculated for each investigator group. For each investigator group, the percentages of correctly diagnosed pathologies, as confirmed by the arthroscopy report, were calculated. Differences in percentage of correct diagnoses among the investigator groups were tested for significance using a Mc Nemar test or exact Mc Nemar test [26]. The threshold for statistical significance was defined at 0.05.

## Results

In total 473 patients were enrolled in the study. Fifty-three patients underwent HA for reasons other than FAIS and were excluded from this study. Furthermore, 148 patients received a dMRA differing from the protocol defined for this study and were also excluded from this study, leaving a total of 272 patients (174 males, 98 females) that met the inclusion criteria and were included in the analysis. Two patients had bilateral dMRA with subsequent HA, yielding 274 hips (104 left hips, 170 right hips). The average patient age at the time of surgery was 39.9 ± 11.8 (18–75) years (Table 2).

**Table 2:** Patient demographic information

	Value
Total no. of patients	272 (274 hips)
Laterality, *n* (%)	
Left	104 (38.0)
Right	170 (62.6)
Sex, *n* (%)	
Male	174 (64)
Female	98 (36)
Age, years	39.9 ± 11.8 (18–75)

Values are shown as the mean ± SD (range)

### Surgical results

The intraoperative findings of all 274 operated hips were evaluated using the surgical reports and intraoperative photo documentation. The incidence of labral pathologies and acetabular chondral damage was 79% (218 of 274) and 69% (190 of 274), respectively. No femoral chondral damage was detected in these patients. The intraoperatively detected incidence and classification of acetabular chondral lesions and labral pathologies are presented in Table 3.

**Table 3:** Surgical findings

	*n* (%)
Labral pathologies (Beck [22])	218(79)
Grade 0	58 (21)
Grade 1 (degeneration)	54 (19.6)
Grade 2 (full-thickness tear)	46 (16.7)
Grade 3 (detachment)	108 (39.1)
Grade 4 (ossification)	10 (3.6)
Acetabular chondral defects (ALAD [21])	192 (69)
Grade 0	86 (31.1)
Grade 1	58 (21.0)
Grade 2	32 (11.6)
Grade 3	62 (22.5)
Grade 4	38 (13.8)

*ALAD* acetabular labrum articular disruption

### Direct MR arthrography (dMRA) results

**Fig. 1:**
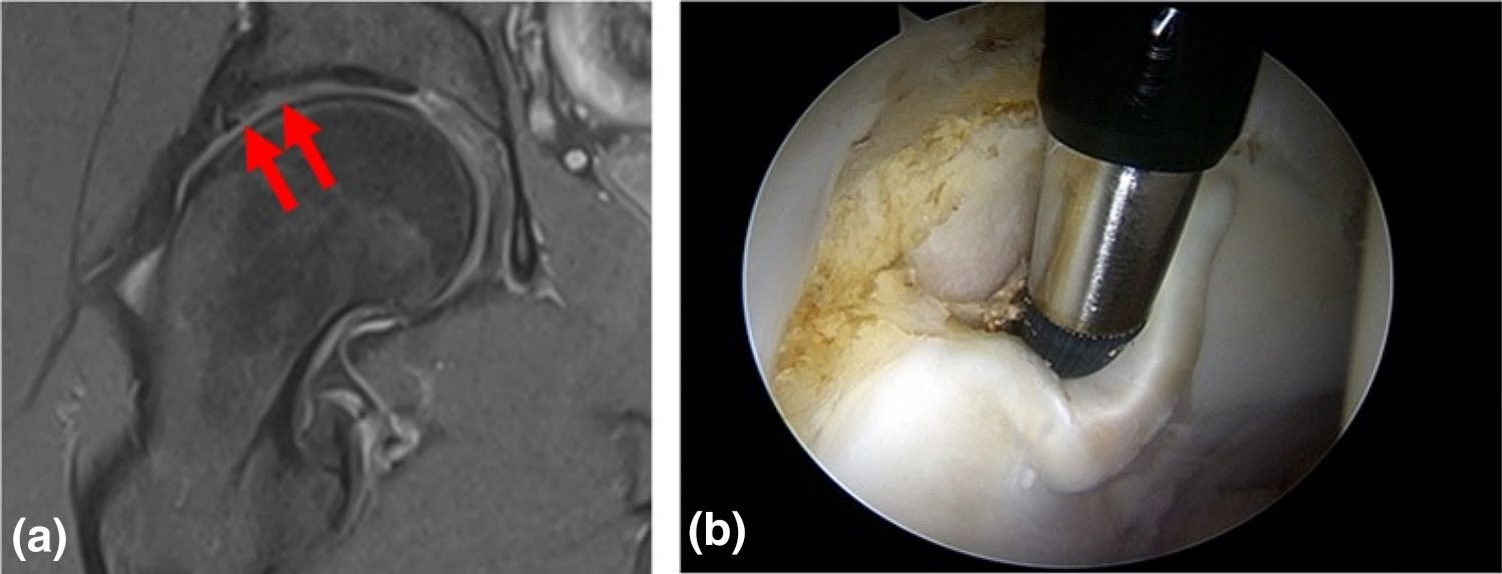
A 24-year-old male. **a** Coronal fat-saturated TSE dMRA image of the right hip demonstrates acetabular chondral delamination (red arrows). **b** Intraoperative finding confirming acetabular chondral flap. This was only recorded as a true-positive result by the hip-arthroscopy trained orthopaedic surgeon

**Fig. 2:**
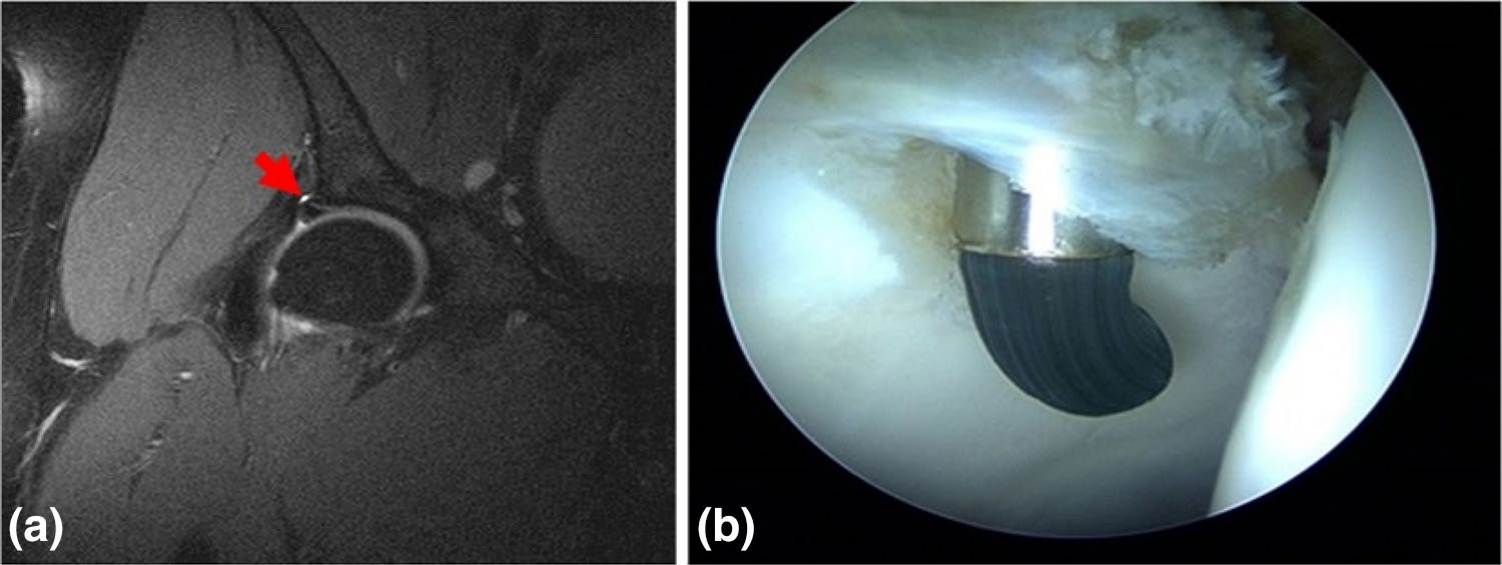
A 27-year-old female. **a** Coronal fat-saturated TSE dMRA image of the right hip reveals a full labrum lesion (Czerny IIIa) (red arrows). **b** Intraoperative finding confirming acetabular labrum lesion (Beck 3). This was recorded as a true-positive result by the hip-arthroscopy trained orthopaedic surgeon and the fellowship-trained musculoskeletal radiologist

**Fig. 3:**
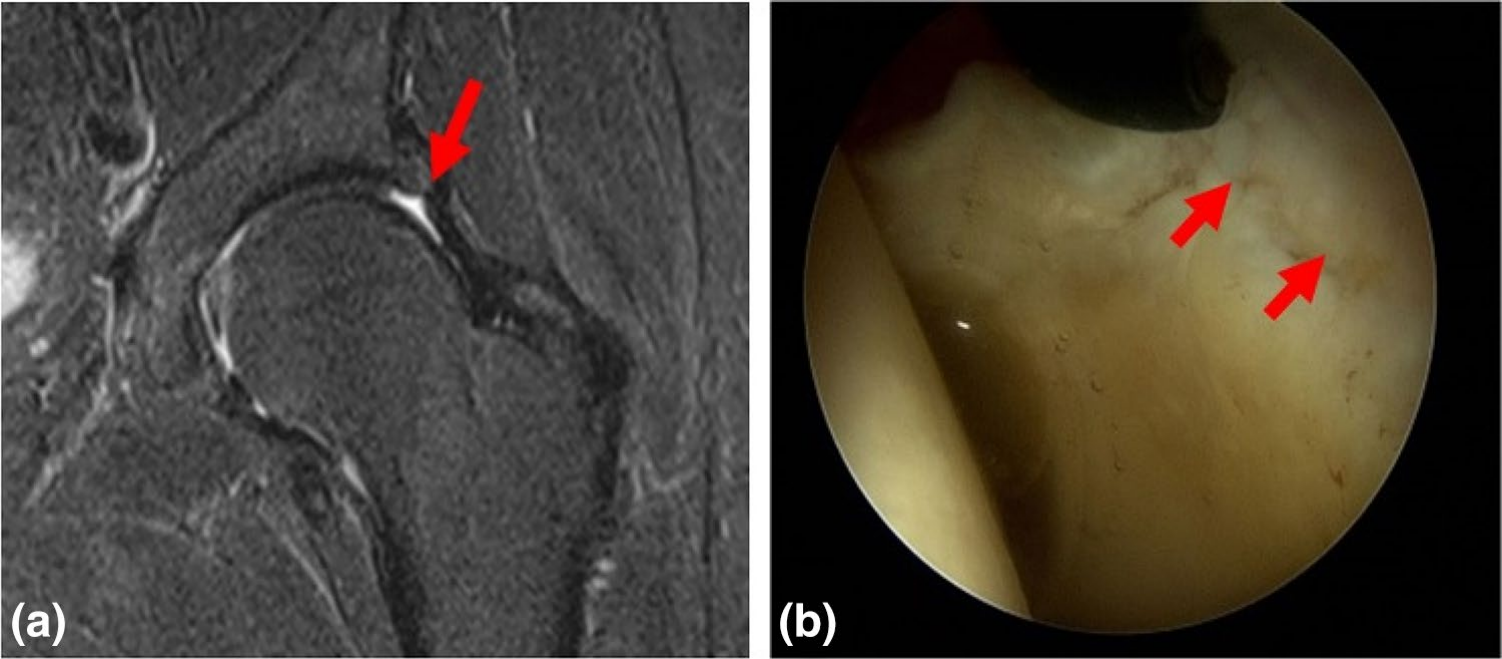
A 34-year-old male. **a** Coronal fat-saturated TSE dMRA image of the left hip reveals a full-thickness acetabular chondral damage (Outerbridge IV) (red arrows). **b** Intraoperative finding confirming full-thickness acetabular chondral damage (ALAD 4). This was recorded as a true-positive result by all investigators

All dMRAs were evaluated independently and in a blinded fashion by the six investigators (three investigator groups). We found very good intraobserver agreement for the three investigator groups (Group 1, ICC 0.91, Group 2, ICC 0.87, Group 3, ICC 0.95). The calculated sensitivity, specificity, PPV, NPV and accuracy values for labral pathologies and acetabular chondral lesions are shown in Tables 4 and 5. The percentages of correctly diagnosed pathologies confirmed by the arthroscopy report are summarized in Table 6. As marked in Table 6, investigator group 3 displays the highest percentage of correctly diagnosed labral pathologies and acetabular chondral lesions, which is significantly higher than the other two investigator groups (*p* = 0.006; Mc Nemar’s test). The results between the investigator groups 1 and 2 did not differ significantly (*p* = 0.575; Mc Nemar’s test). Figures 1, 2, 3 show examples of MRI findings that were evaluated differently by the investigators.

**Table 4:** Comparison of the mean values of three investigator groups for evaluating labral pathologies

	Sensitivity (95% CI)	Specificity (95% CI)	PPV (95% CI)	NPV (95% CI)	Accuracy (95% CI)
Investigator group 1	79% (73–84)	18% (9–31)	79% (76–81)	18% (10–29)	66% (60–72)
Investigator group 2	83% (77–88)	36% (23–50)	83% (80–86)	36% (26–47)	74% (68–79)
Investigator group 3	88% (83–92)	53% (40–67)	88% (95–91)	54% (43–64)	81% (76–85)

*PPV* positive predictive value, *NPV* negative predictive value

**Table 5:** Comparison of the mean values of the three investigator groups for evaluating acetabular chondral lesions

	Sensitivity (95% CI)	Specificity (95% CI)	PPV (95% CI)	NPV (95% CI)	Accuracy (95% CI)
Investigator group 1	81% (75–86)	36% (26–46)	71% (67–74)	50% (40–60)	66% (60–71)
Investigator group 2	84% (78–89)	38% (28–49)	75% (72–79)	52% (41–62)	70% (64–75)
Investigator group 3	91% (87–95)	51% (40–62)	81% (77–84)	73% (62–82)	79% (74–84)

*PPV* positive predictive value, *NPV* negative predictive value

**Table 6:** Percentage of correctly diagnosed pathologies confirmed by arthroscopy for the three investigator groups

Pathology	Correct diagnosis (%)		
	Investigator group 1	Investigator group 2	Investigator group 3
Labral pathologies	79	83	93*
			*p* = 0.006 vs. Investigator group 1 and 2
Acetabular chondral lesions	79	82	89*
			*p* = 0.006 vs. Investigator group 1 and 2

^*^Mc Nemar’s test for paired proportions; *p* < 0.05

## Discussion

The interpretation of preoperative imaging is an important factor to adequately target the preoperative planning of an impending surgical procedure. This is the first study investigating the influence of the level of experience and comparing the accuracy of evaluating dMRA of the hip joint between orthopaedic surgeons with different experiences in HA and radiologists. The most important finding of this study is that HA-trained orthopaedic surgeons achieve significantly better evaluation of dMRA images compared to non-HA-trained orthopaedic surgeons or fellowship-trained musculoskeletal radiologists. These results are significant because an HA-trained orthopaedic surgeon should independently assess the MRI examination to best discuss the MRI results with the patient and the appropriate expected outcomes.

DMRA is the gold standard for visualization of intraarticular hip pathologies and has been proven superior to cMRI and indirect MRA (iMRA) in a meta-analysis by Saied et al.[27]. Recent studies have shown that the severity of chondral damage significantly influences the postoperative outcome after HA [28–30]. Therefore, it is essential that MRI images are correctly interpreted to properly discuss possible treatment options with the patient and not raise false expectations. It has been shown that patient satisfaction is related to preoperative expectations, for example, in patients undergoing arthroplasty [31]. Consequently, an adequate interpretation of the preoperative diagnostics with straightforward physician–patient communication is necessary to unrealistic expectations and, thus, dissatisfaction with surgery.

The sensitivities in our study for the detection of labral pathologies and acetabular chondral lesions varied between 79 and 88% and 81% to 91%, respectively, for the three investigator groups. These values are consistent with previously reported sensitivities for detecting acetabular labral pathologies and chondral lesions by experienced musculoskeletal radiologists [10, 32]. Nevertheless, HA-trained orthopaedic surgeons achieved better evaluation of dMRA images regarding the sensitivities for detecting labral and acetabular chondral lesion.

The phenomenon that a specialist orthopaedic surgeon achieves higher accuracies for detecting pathologies in MR imaging than a specialist radiologist is also found in the literature for other joints [19, 33]. It has been shown that experienced orthopaedic surgeons are more accurate than radiologists in assessing traumatic anterior shoulder instability–related lesions on MRA or in assessing shoulder impingement [19, 33]. These results, although dealing with joints other than the hip, confirm our findings. Since a specialized orthopedic surgeon often deals with only one joint, he achieves a higher degree of specialization than a specialized radiologist, who often covers the entire field of musculoskeletal radiology. To improve the quality of future radiological reports, it is important for the HA-trained orthopaedic surgeon and musculoskeletal radiologist to establish a personal dialog and to communicate between the two disciplines. Intensified collaboration between orthopaedic surgeons and radiologists with direct feedback can improve diagnostic performance, which will improve future diagnostics including earlier detection of crucial pathologies and treatment.

One point for consideration in the evaluation of MR images is the occurrence of norm variants. For example, the supraacetabular fossa can simulate acetabular cartilage damage in MRI [34]. The same applies to the physiological sublabral recess, which can be falsely interpreted as a ruptured labrum [35]. These norm variants can lead to false-positive radiological reports. An experienced orthopedic surgeon can draw conclusions from the intraoperative findings to the MRI reports and thus better evaluate such norm variations in future MRIs. Overall, the medical history, clinical examination and imaging should be consistent to make the correct diagnosis. The surgeon’s experience plays an important role in evaluating dMRA, as experienced surgeons can assess the significance of, for example, delamination of the acetabular cartilage more reliably. Based on knowledge of intraoperative findings, experienced surgeons are aware of these pathologies and can adequately identify them in dMRA.

Our study is not free of limitations. First, the average time delay between dMRA and hip arthroscopy was 4.5 months. Therefore, it is theoretically possibility that the labral pathologies and chondral lesions occurred or worsened during the interval between dMRA imaging and HA. Additionally, our study evaluated 1.5 T dMRA images and not 3 T dMRA images. Recent studies have reported higher sensitivities for detecting labral pathologies or chondral lesions in 3 T dMRAs [15, 32]. However, a 3 T MRI is not representative of standard radiological imaging in Germany, as 1.5 T MRIs are more widely available and represent the current standard for MRI imaging of the hip throughout the country. Furthermore, it must be noted that the inter-reliability of an intra-articular examination during hip arthroscopy may be questionable, as surgeons may assess pathologies differently. However, the analysis of inter-reliability between two or more surgeons was not part of the present study.

## Conclusion

The most important finding of this study is that the accuracy of dMRA in detecting labral pathologies or acetabular chondral lesions depends on the examiner and his level of experience in hip arthroscopy. The highest values are found for HA-trained orthopaedic surgeons compared to non-HA-trained orthopaedic surgeons or fellowship-trained musculoskeletal radiologists. As opposed to a HA-trained orthopaedic surgeon, a non-HA-trained orthopaedic surgeon may consult the results from a musculoskeletal trained radiologist to help in making a diagnosis with dMRI.
